# The influence of a first-order antedependence model and hyperparameters in BayesCπ for genomic prediction

**DOI:** 10.5713/ajas.18.0102

**Published:** 2018-07-26

**Authors:** Xiujin Li, Xiaohong Liu, Yaosheng Chen

**Affiliations:** 1 State Key Laboratory of Biocontrol, School of Life Sciences, Sun Yat-sen University, North Third Road, Guangzhou Higher Education Mega Center, Guangzhou, Guangdong 510006, China

**Keywords:** BayesCπ, Antedependence Model, Hyperparameter

## Abstract

**Objective:**

The Bayesian first-order antedependence models, which specified single nucleotide polymorphisms (SNP) effects as being spatially correlated in the conventional BayesA/B, had more accurate genomic prediction than their corresponding classical counterparts. Given advantages of BayesCπ over BayesA/B, we have developed hyper-BayesCπ, ante-BayesCπ, and ante-hyper-BayesCπ to evaluate influences of the antedependence model and hyperparameters for v_g_ and ${\text{s}}_{\text{g}}^{2}$ on BayesCπ.

**Methods:**

Three public data (two simulated data and one mouse data) were used to validate our proposed methods. Genomic prediction performance of proposed methods was compared to traditional BayesCπ, ante-BayesA and ante-BayesB.

**Results:**

Through both simulation and real data analyses, we found that hyper-BayesCπ, ante-BayesCπ and ante-hyper-BayesCπ were comparable with BayesCπ, ante-BayesB, and ante-BayesA regarding the prediction accuracy and bias, except the situation in which ante-BayesB performed significantly worse when using a few SNPs and π = 0.95.

**Conclusion:**

Hyper-BayesCπ is recommended because it avoids pre-estimated total genetic variance of a trait compared with BayesCπ and shortens computing time compared with ante-BayesB. Although the antedependence model in BayesCπ did not show the advantages in our study, larger real data with high density chip may be used to validate it again in the future.

## INTRODUCTION

Methods for genomic prediction have been developed widely since Meuwissen et al [1] firstly proposed genomic selection methods. To date, there are mainly two classical types of methods applied to genomic prediction in livestock breeding. The first one is BLUP methods such as GBLUP [2], which assume that single nucleotide polymorphisms (SNP) effects independently and identically follow a normal distribution with an equal variance. The second one is Bayesian hierarchical models with different prior distributions on the variances of SNP effects. For example, BayesA assumes a scaled-inverted chi square distribution on SNP-specific variances [1].

Habier et al [3] firstly developed BayesCπ for genomic prediction to address the drawbacks of BayesA and BayesB with respect to influences of prior hyperparameters and the prior probability π that a SNP has zero effect. BayesCπ assumes that all SNPs have a common effect variance instead of locus-specific variances in BayesA or BayesB so that the influence of ${\text{s}}_{\text{g}}^{2}$ becomes smaller. π = 0.95 or 0.99 in BayesB is generally arbitrarily defined. It is improved in BayesCπ where π is treated as an unknown being inferred from the data. Although the statistical drawbacks of BayesA and BayesB did not impair the prediction accuracy mainly due to linkage disequilibrium (LD) information, accounting for reducing computing time and better fitting the genetic architecture of a trait, BayesCπ can be deemed to have better merit for routine application compared with BayesA/B.

So far, most of Bayesian methods, such as BayesA, BayesB, and BayesCπ, have a common feature that SNP effects are assumed to be independent. However, the existence of LD between SNPs makes the above assumption improper, and a close relationship should exist between SNPs. To account for LD between SNPs, Yang and Tempelman [4] brought first-order antedependence models which model SNP effects as correlated into the conventional BayesA and BayesB, and developed ante-BayesA and ante-BayesB. It is reported in their paper that the antedependence methods had significantly higher accuracies than the corresponding classical counterparts at higher LD levels (r^2^>0.24) in the simulation study, and they were also more accurate in mice data and other benchmark data sets.

However, although there are advantages of BayesCπ over BayesA and BayesB aforementioned, first-order antedependence models are still not brought to BayesCπ. Thus, we will develop ante-BayesCπ, and then investigate whether ante-BayesCπ can improve the prediction accuracy and bias compared to BayesCπ. Besides, it has not been widely appreciated that v_g_ and ${\text{s}}_{\text{g}}^{2}$ also can be estimated from the data [5]. This recognition is important as both hyperparameters can help define the genetic architecture of a trait of interest. The impact of adding hyperparameters to BayesCπ for genomic prediction is little reported and is also an interesting topic for investigation.

Therefore, the objective of this study was to investigate the impact of adding a first-order antedependence model and hyperparameters into BayesCπ for genomic prediction regarding the prediction accuracy and bias. We have demonstrated the predictive ability of BayesCπ with first-order antedependence models and hyperparameters in simulation data from the study of Jiang et al [6], the 15th quantitative trait loci-marker assisted selection (QTL-MAS) workshop data set and the heterogeneous stock mice, compared to the classical BayesCπ, ante-BayesA, and ante-BayesB.

## MATERIALS AND METHODS

### Statistical model

The general linear mixed model used for genomic prediction could be written as

(1) y=Xβ+Zg+e

Here, y was a vector of phenotypes, β was a vector of fixed effects, g was a vector of random SNP effects, and e was a residual vector. X was a known incidence matrix linking β to y. Z was a known matrix linking g to y in which SNP genotypes were coded as 0, 1, or 2 copies of one allele for each SNP (column) and animal (row). Furthermore, we specified **g** ~N (0,**G**), where $G=\text{diag}(\sigma_{g_{j}}^{2})$ and $e\sim \text{N }(0,I\sigma_{e}^{2})$.

### BayesCπ

Habier et al [3] firstly developed BayesCπ, and the obvious difference between BayesCπ and other methods such as GBLUP, BayesA, and BayesB was the characterization of **G**. $\sigma_{g_{j}}^{2}$ in BayesCπ had a two-component mixture with one component being 0 and the other component being one fixed value $\sigma_{g}^{2}$, i.e.,

(2) $$\sigma_{g_{i}}^{2}|v_{g},s_{g}^{2},\pi \;\{\begin{array}{ll} =0 & \text{with probability }\pi \\ =\sigma_{g}^{2}\sim x^{-2}(v_{g},\;s_{g}^{2}) & \text{with probability }1-\pi \end{array}$$

Here, π represented the proportion of SNPs having no genetics effects on the trait of interest. Based on Habier et al [3], the prior of π was treated as an unknown with uniform (0,1). The v_g_ was set to 4.2, and ${\text{s}}_{\text{g}}^{2}$ was set as follows:

(3) $${\text{s}}_{\text{g}}^{2}=\frac{{\tilde{\sigma }}_{\text{g}}^{2}({\text{v}}_{\text{g}}-2)}{{\text{v}}_{\text{g}}}$$

(4) $${\tilde{\sigma }}_{\text{g}}^{2}=\frac{{\tilde{\sigma }}_{\text{a}}^{2}}{\left(1-\pi )\Sigma_{\text{k}=1}^{\text{K}}2{\text{p}}_{\text{k}}(1-{\text{p}}_{\text{k}}\right)}$$

Where ${\tilde{\sigma }}_{\text{g}}^{2}$ was the additive genetic variance for a SNP, and ${\tilde{\sigma }}_{\text{a}}^{2}$ was the additive genetic variance explained by all SNPs.

### Hyper-BayesCπ

We labeled hyper-BayesCπ when we sampled v_g_ using a Metropolis Hastings (MH) update and ${\text{s}}_{\text{g}}^{2}$ with a Gibbs update in BayesCπ. Similar to BayesA and BayesB, we specified the following prior distributions on v_g_ and ${\text{s}}_{\text{g}}^{2}$ for hyper-BayesCπ: v_g_~p(v_g_) ∝ (v_g_+1)^−2^, and ${\text{s}}_{\text{g}}^{2}\sim Gamma(\alpha_{s},\beta_{s})$ with shape parameter *α_s_* =1.0 and rate parameter *β_s_* =1.0. The full conditional density (FCD) of v_g_ did not have a recognizable form, and a random walk MH step other than a Gibbs step was used to sample v_g_ from this FCD. The detailed procedure can be seen Yang et al [7]. The FCD of ${\text{s}}_{\text{g}}^{2}$ had a recognizable form with a Gamma distribution, provided that a conditionally conjugate Gamma prior is used [8].

### Ante-BayesCπ

Ante-BayesCπ was defined here as combining BayesCπ and the first-order antedependence model proposed from the study of Yang and Tempelman [4]. Likewise, the following nonstationary first-order antedependence correlation structure for **g** based on the relative physical location of SNPs along the chromosome was used:

(5) $${\text{g}}_{\text{j}}=\{\begin{array}{cc} \delta_{1} & \text{if j}=1\\ {\text{t}}_{\text{j},\text{j}-1}{\text{g}}_{\text{j}-1}+\delta_{\text{j}} & \text{if }2\leq \text{j}\leq \text{m} \end{array}$$

Here, t_j,j-1_ was the marker interval-specific antedependence parameter of g_j_ on g_j–1_ in the specified order. δ_j_ was the residual SNP effect following $\text{N}(0,\sigma_{\delta_{\text{j}}}^{2})$. Different from ante-BayesA and ante-BayesB, $\sigma_{\delta_{\text{j}}}^{2}$ in ante-BayesCπ had a two-component mixture prior with the following definition:

(6) $$\sigma_{\delta_{\text{i}}}^{2}|{\text{v}}_{\delta },{\text{s}}_{\delta }^{2},\pi \{\begin{array}{ll} =0 & \text{with probability }\pi \\ =\sigma_{\delta }^{2}\sim {\text{x}}^{-2}({\text{v}}_{\delta },\;{\text{s}}_{\delta }^{2}) & \text{with probability }1-\pi \end{array}$$

It should be noted that, here π represented the proportion of SNPs having no residual SNP effects, which was a little different from π of BayesCπ and hyper-BayesCπ.

### Ante-hyper-BayesCπ

We labeled the model as ante-hyper-BayesCπ in which the hyperparameters for v_g_ and ${\text{s}}_{\text{g}}^{2}$ and a nonstationary first-order antedependence correlation structure for **g** were combined.

For ante-BayesCπ and ante-hyper-BayesCπ, we specified ${\text{t}}_{\text{j},\text{j}-1}\sim N(u_{t},\sigma_{t}^{2})$ as a conjugate prior, and further $u_{t}\sim N(u_{t0},\sigma_{t0}^{2})$ and $\sigma_{t}^{2}\sim x^{-2}(v_{t},s_{t}^{2})$ where *u_t_*_0_, $\sigma_{t0}^{2}$, *v_t_*, and $s_{t}^{2}$ were specified to be known [4]. It should be noted that t_j,j-1_ was set 0 between the last SNP of one linkage group or chromosome and the first SNP in the subsequent group. The remaining priors were also specified, such as π~ U(0,1), v_δ_ = v_g_ and ${\text{s}}_{\delta }^{2}={\text{s}}_{\text{g}}^{2}$ in ante-BayesCπ, ${\text{s}}_{\delta }^{2}\sim Gamma(\alpha_{s},\beta_{s})$ and v_δ_~p(v_δ_)∝(v_δ_+1)^−2^ in ante-hyper-BayesCπ. For all methods, the noninformative prior $\sigma_{e}^{2}$ is specified, i.e., $\sigma_{e}^{2}\sim x^{-2}(-1,0)$, which is congruent with specifying uniform prior on $\sigma_{e}^{2}$, and in line with recommendations for variance components by Gelman [9]. The full conditional densities of all unknown parameters and any necessary MH steps using Markov chain Monte Carlo are further provided in studies of Habier et al [3] and Yang and Tempelman [4].

We used Fortran 90 to compile BayesCπ, extent forms of BayesCπ, ante-BayesA and ante-BayesB, and program codes can be seen in Supplementary Material (See e-version for Supplement). It should be pointed out that ante-BayesB with a pre-defined π in our paper was extended from BayesB of Meuwissen et al [1], while π was inferred from the data in ante-BayesB of Yang and Tempelman [4].

In order to evaluate the performance of our proposed methods, we used data sets from other reported studies, i.e., simulation data from the study of Jiang et al [6], the 15th QTL-MAS workshop data set and real data on the heterogeneous stock mice from studies of Yang and Tempelman [4] and Gao et al [10], to compare them with ante-BayesA and ante-BayesB with respect to the accuracy and bias of genomic prediction.

### Simulation data

We downloaded simulation data from the study of Jiang et al [6], and the detailed simulation process could be seen in their study. Briefly, the default scenario was as follows: a total of 30 QTLs with a minor allele frequency >0.05 was randomly selected, and their effects on two traits were drawn from a standard bivariate normal distribution with correlation 0.5. Normal error deviates were added to achieve heritablities of 0.5 for trait 1 and 0.1 for trait 2. The error covariance between two traits was set to 0.

Sampling every 10th and 25th SNPs from the full set of SNPs respectively was conducted to form two subsets of SNPs. Averaged over 30 replicates, the average number of SNPs were 4,119, 411, and 164 for the full set and two subsets, which resulted in three average LD levels (r^2^ = 0.33, 0.22, and 0.14) of adjacent SNPs corresponding to the full set of SNPs and other two subsets with SNP intervals of 10 and 25, respectively. In our study, we selected trait 1 with both phenotype and genotype for first 20 of 30 replicates with the full dataset and other two subsets of SNPs as test data to investigate the influence of LD between adjacent SNPs on the prediction accuracy and bias. Generation 5001 was considered as the reference population, and generation 5002 as the candidate population.

### Analysis of the 15th QTL-MAS workshop data set

A base population was a collection of 20 sires and 200 dams. Each sire was mated to 10 dams, and each dam was mated to only one sire. Within each family, one dam gave birth to 15 offspring. The 10 progenies were randomly assigned to the reference population with trait phenotypes and marker genotypes information, and other 5 belonged to the candidate population, only recorded for genotype marker information. Thus, 2000 individuals formed the reference population, and the candidate population included 1,000 individuals. A genome consisting of 9,990 SNPs on five chromosomes with 1 Morgan each were simulated without any missing data and genotyping error. Eight QTLs (1 quadri-allelic, 2 linked in phase, 2 linked in repulsion, 1 imprinted and 2 epistatic) were simulated. Random error was added to create an heritability of 0.30 for the trait analyzed [11]. In the following analyses, we removed all SNPs with minor allele frequency = 0, leaving 7,121 SNPs on five chromosomes for comparing performances of different Bayesian methods aforementioned. These SNPs had been sorted by the physical position.

### Application to heterogeneous stock mice data set

A population of heterogeneous stock mice was generated by the Wellcome Trust Centre for Human Genetics (WTCHG) (http://gscan.well.ox.ac.uk/). This population was formed by a crossing of eight inbred strains, followed by 50 generations of pseudorandom mating [12]. The extent of LD in this population is small, and average r^2^ among adjacent SNPs is 0.62 [13]. It is well known for the family structure and history of this population, and thus interpretation of results will be easy.

In order to compare performances of genomic prediction using different Bayesian methods, we selected four traits: body weight at 6 weeks (W6W), growth slope between 6 and 10 weeks of age (GSL), body mass index (BMI), and body length (BL) similar to the study of Legarra et al [13]. For computational simplicity, the pre-corrected phenotypes of these traits provided by Valdar et al [14] were used as pseudo phenotypes for the following analysis. Two sets of genotype information were used, which were directly from studies of Yang and Tempelman [4] and Gao et al [10]. Genotypic data processing of Yang and Tempelman [4] resulted in the data set involving records on 1,917 animals with 950 SNPs on the 19 chromosomes, and the average LD of r^2^ was 0.35 between adjacent markers. In the study of Gao et al [10], there were 1,940 animals and 9,266 SNPs on the 19 chromosomes after their quality control steps, which resulted in the average LD of r^2^ about 0.60.

Numbers of animals having both phenotypes and two sets of genotypes were 1,917, 1,901, 1,814, and 1,821 for W6W, GSL, BMI, and BL respectively. All animals for each trait were randomly divided into two nearly equal-sized partitions of reference and candidate data sets. This was replicated 20 times for comparison of genomic prediction among different Bayesian methods. SNPs were sorted based on their physical positions along the chromosome.

For each of the six Bayesian methods, ante-BayesA, ante-BayesB, BayesCπ, hyper-BayesCπ, ante-BayesCπ, and ante-hyper-BayesCπ, in both simulation data and real data on the heterogeneous stock mice, the Markov chains were run for 350,000 cycles of Gibbs sampling (for ant-BayesB, 100 additional cycles of MH sampling cycle), and the first 50,000 cycles were discarded as the burning period. After the burning period, every 10th cycles were subsequently saved for obtaining estimates of SNP effects. In ante-BayesB, we set π = 0.95 in both the simulation data of Jiang et al [10] and the heterogeneous stock mice application, and 0.99 in the 15th QTL-MAS workshop data set.

Direct genomic values (DGVs) for individuals with genotypes, but no phenotypes, were calculated as the sum of all SNP effects according to their SNP genotypes. The prediction accuracy was calculated as Pearson’s correlation between DGVs and true breeding values (TBVs) in simulation data (or pre-corrected phenotypes in mice data) for the candidate population, and the prediction bias was evaluated by the regression coefficient of DGVs on TBVs in simulation data (or pre-corrected phenotypes in mice data). For the simulation data of Jiang et al [10] and the heterogeneous stock mice, one-way analysis of variance was performed to determine the statistical significance of differences in the accuracy of genomic prediction among above six methods and in estimates of π among BayesCπ, hyper-BayesCπ, ante-BayesCπ and ante-hyper-BayesCπ. The stringent Bonferroni multiple test corrections were used.

## RESULTS

### Results from the simulations

The prediction accuracies and unbias under three different LD levels can be seen in Table 1. For the prediction accuracy, there are not the statistical significance of differences in the accuracy of genomic prediction among six methods for all scenarios (p>0.05). Hyper-BayesCπ performed a little better or similar among all six methods in three scenarios of different LD levels. In the scenario of using all SNPs, ante-hyper-BayesCπ performed as well as hyper-BayesCπ, which was 0.6% higher than BayesCπ and ante-BayesCπ, 0.5% for ante-BayesB, and 0.8% for ante-BayesA, respectively. In other two scenarios, ante-hyper-BayesCπ and ante-BayesCπ performed a little worse than the counterparts. The prediction accuracy for ante-BayesB was much lower than other methods when using the SNP data set with the interval of 25 SNPs which was close to the level of significant difference (p = 0.07).

Regarding the prediction bias, the regression coefficients for all methods were close to 1.0, which indicated good unbiased prediction. Additionally, although there were similar prediction accuracies among BayesCπ and extensions of BayesCπ, the estimated π value from BayesCπ were significantly lower than those from extensions of BayesCπ in the scenario of using all SNPs, ante-(hyper-) BayesCπ had significantly lower π value than (hyper-) BayesCπ in the scenario of the interval of 10 SNPs, and the estimates of π were comparable in the scenario of the interval of 25 SNPs. We found that the π value became smaller as the density of SNPs decreased (Table 2).

### Common data set of the 15th QTL-MAS workshop

Using the above six methods, we also analyzed the 15th QTL-MAS workshop data set. As shown in Table 3, ante-hyper-BayesCπ and ante-BayesCπ had the same prediction accuracy (0.942), and they were 0.1%, 0.3%, 0.6%, and 1.2% higher than hyper-BayesCπ, BayesCπ, ante-BayesB, and ante-BayesA, respectively. The regression coefficients ranged from 1.044 to 1.068 indicating the unbiased genomic prediction for all methods. The π values estimated from BayesCπ, hyper-BayesCπ, ante-BayesCπ, and ante-hyper-BayesCπ were very similar which were 0.996, 0.997, 0.996, and 0.997, respectively.

### Real heterogeneous stock mice data set

As shown in Table 4, the genomic prediction for all six methods was comparable for all traits except for W6W in the scenario of low density SNP data set. The ante-BayesB using low density SNP data performed worse than other methods for all traits (statistical significance for W6W). The hyper-BayesCπ and ante-hyper-BayesCπ sometimes performed a little better than BayesCπ and ante-BayesCπ, such as for body weight trait. Using SNP data set from low density to high density did not reflect the advantage of ante-hyper-BayesCπ (ante-BayesCπ) over hyper-BayesCπ (BayesCπ) on the prediction accuracy for these four trait analyzed in mice data. Additionally, all the methods gave unbiased genomic prediction for each trait, which can be seen in Table 5.

As shown in Table 6, the estimated π were very different for different traits due to different genetic architectures of different traits. The estimated π values ranged from 0.327±0.010 to 0.949±0.009. The estimated π for W6W were higher than other three traits. The estimates of π from BayesCπ, hyper-BayesCπ, ante-BayesCπ, and ante-hyper-BayesCπ were significantly different (p<0.05) for all scenarios except for BMI with high density SNP data.

## DISCUSSION

Our study is the first one to comprehensively investigate the influence of a first-order ante-dependence model and key hyperparameters on BayesCπ using simulation data sets and real mice data set. According to our results, the prediction accuracies for hyper-BayesCπ, ante-BayesCπ, and ante-hyper-BayesCπ were comparable with BayesCπ, ante-BayesB and ante-BayesA except the situation where ante-BayesB performed worse when using a few SNPs and π = 0.95. BayesCπ with an antedependence model and hyperparameters did not work better than BayesCπ regarding the prediction accuracy and bias. Meanwhile, ante-(hyper-) BayesCπ had a longer computing time than BayesCπ and hyper-BayesCπ, and there were similar computing times between BayesCπ and hyper-BayesCπ. When SNP density increased, computing time for ante-(hyper-) BayesCπ increased faster than (hyper-) BayesCπ.

BayesA, BayesB, BayesCπ and their extended forms assume that the prior distribution of variances of SNP non-zero effects is a scaled inverse chi-square distribution with degree of freedom v_g_ and a scale parameter ${\text{s}}_{\text{g}}^{2}$ [15]. Preliminarily, v_g_ and ${\text{v}}_{\text{g}}{\text{s}}_{\text{g}}^{2}$ were fixed to 4.01 and 0.0020 in BayesA, and 4.23 and 0.0429 in BayesB in the first genomic prediction paper of Meuwissen et al [1]. However, it is more reasonable that these two fixed hyperparameters are assigned as different values in different genetic architectures of traits of interest. In the study of Habier et al [3], ${\text{s}}_{\text{g}}^{2}$ was calculated using formula (3) and (4) with different genetic variances for different traits in BayesA, BayesB, and BayesCπ. Many studies have also proposed some alternative priors to estimate these two hyperparameters. Habier et al [3] proposed BayesDπ treating ${\text{s}}_{\text{g}}^{2}$ as unknown with Gamma (1, 1). Yi and Xu [8] developed a model to assign a uniform density on 1/v_g_ for the range (0,1) and a uniform distribution on ${\text{s}}_{\text{g}}^{2}$ for the range (0, A) with A being a large number in the extent of BayesA. Yang and Tempelman [4] used a Gamma (0.1, 0.1) prior distribution on ${\text{s}}_{\text{g}}^{2}$ and specified v_g_~p(v_g_)∝(v_g_+1)^−2^ for both BayesA and BayesB. In our study, we have applied

v_g_~p(v_g_)∝(v_g_+1)^−2^ for v_g_ and a Gamma (1,1) prior distribution on ${\text{s}}_{\text{g}}^{2}$ in BayesCπ, termed as hyper-BayesCπ. The results from our analysis showed that hyper-BayesCπ performed a little better than BayesCπ regarding prediction accuracy and bias. Given needing a pre-estimated genetic variance to define in BayesCπ, hyper-BayesCπ is more feasible to be applied in livestock breeding compared with BayesCπ.

Compared with hyper-BayesCπ (BayesCπ), ante-hyper-BayesCπ (ante-BayesCπ) did not show advantages in improving the prediction accuracy and bias for simulation data with LD levels r^2^ ranging from 0.136 to 0.333 and mice data with two different LD levels. This phenomenon is very different from the performance of ante-BayesA (ante-BayesB) over BayesA (BayesB) in the study of Yang and Tempelman [4]. They reported that ante-BayesA and ante-BayeB had significantly higher accuracies than their corresponding classical counterparts at higher LD levels in simulation data and real small data. However, subsequent studies did not show that the antedependence model performed significantly better. Wang et al [16] evaluated the antedependence model performance in Danish pigs and found that ante-BayesA showed lower accuracy compared to other models. Jiang et al [6] also introduced the first-order antedependence model to multi-trait BayesA, and the analysis from simulation and mice data showed that multi-trait ante-BayesA had less than 1% higher accuracies than multi-trait BayesA. The results from these studies were similar to the performance of ante-hyper-BayesCπ (ante-BayesCπ) over hyper-BayesCπ (BayesCπ) from our study.

When using genotype data from low density to high density in mice data, the antedependence model was not more advantageous over the corresponding method for four traits with different heritabilities. This surprising phenomenon may result from the following reasons. On one hand, the antedependence model had higher number of effective parameters, which suggested that the accuracy estimating SNP effects may be poor in current data set (less than 1,000 individuals in reference population) due to model complexity [16]; On the other hand, the relationship for adjacent SNPs is more closed with increasing SNP density. The linear relationship may not be good to explain the relationship for adjacent SNPs, and non-linear relationship may be better. In the future, with sequence data widely being used, the performance of the antedependence model can be validated again in livestock with sequence data.

Habier et al [3] reported that estimates of π from BayesCπ were sensitive to training data size and SNP density. From our results, estimates of π not only were sensitive to SNP density but also were sensitive to the genetic architecture of a quantitative trait. Compared with ante-BayesA and ante-BayesB, BayesCπ and its extensions could provided more information about the genetic architecture of a trait of interest. Although different estimates of π from BayesCπ and its extensions led to little differences on the prediction accuracy, they may have different power for QTL mapping which is interesting to be further studied. Additionally, it should be noted that ante-BayesB in this study was extended from the classical BayesB proposed by Meuwissen et al [1]. Ante-BayesB performed significantly worse than other methods when using a few SNPs, which suggests that treating π as known with a high value may be a poor choice in some cases. This agrees with Daetwyler et al [17] who reported that GBLUP outperformed BayesC with a fixed π when the number of simulated QTL was large, which is also validated by Habier et al [3]. This may be the reason why the extent of classical BayesB in which π is estimated by using a prior distribution and data information was proposed, such as ante-BayesB developed by Yang and Tempelman [4].

Ante-BayesA, ante-BayesB, and ante-(hyper-)BayesCπ had a similar genomic prediction accuracy, and no one outperformed the other methods across all traits, which is consistent with the study of Habier et al [3] who reported the similar prediction accuracy between BayesA, BayesB, and BayesCπ. However, computing time is very different among these three ante-Bayesian methods. From our study, ante-BayesA is the fastest, ante-(hyper-) BayesCπ is next, and ante-BayesB is the slowest. Ante-BayesB had the longest computing time because the 100 cycles of MH step for sampling the locus-specific variances in the implementation of ante-BayesB is repeated in each iteration. Ante-BayesA and ante-(hyper-) BayesCπ had the advantage on computing time, which becomes more and more important as SNP density increases. Compared with ante-BayesA, ante-(hyper-) BayesCπ can shrink SNP effects and is more sensitive to the genetic architecture of a trait of interest, which results in gaining higher accuracies for traits with some large QTL effects, such as fat yield in Holstein populations [18,19].

## CONCLUSION

In conclusion, BayesCπ with prior distributions on v_g_ and ${\text{s}}_{\text{g}}^{2}$ (assigned as hyper-BayesCπ) is recommended because it has the comparable prediction accuracy and bias and avoids pre-estimated total genetic variance of a trait compared with BayesCπ. The performance of the first-order antedependence model in BayesCπ did not show an advantage in improving the prediction accuracy and bias.

## Supplementary Data



## Figures and Tables

**Table 1:** Accuracies (mean±SE) and biases (mean±SE) of DGVs in the validation population of simulated data sets under different LD levels of adjacent markers over 20 replications

Method	All		Every 10th		Every 25th	
		
	Corr	Reg	Corr	Reg	Corr	Reg
Ante-BayesA	0.873±0.008	1.034±0.024	0.829±0.009	1.028±0.029	0.760±0.010	1.010±0.025
Ante-BayesB	0.876±0.008	1.037±0.025	0.824±0.010	1.065±0.036	0.719±0.014	1.069±0.039
BayesCπ	0.875±0.008	1.044±0.025	0.832±0.008	1.056±0.031	0.760±0.010	1.023±0.025
Hyper-BayesCπ	0.881±0.008	1.025±0.025	0.833±0.008	1.041±0.031	0.760±0.011	1.007±0.024
Ante-BayesCπ	0.875±0.008	1.051±0.026	0.824±0.008	1.110±0.032	0.754±0.011	1.141±0.032
Ante-hyper-BayesCπ	0.881±0.008	1.042±0.025	0.826±0.008	1.103±0.031	0.754±0.011	1.133±0.032

SE, standard error; DGVs, direct genomic values; LD, linkage disequilibrium; Corr, Pearson’s correlation; Reg, regression coefficient.

**Table 2:** π values (mean±standard error) estimated from BayesCπ and extentions of BayesCπ using the reference population of simulated data sets under different LD levels of adjacent SNPs over 20 replications

Method	ALL	Every 10th	Every 25th
BayesCπ1)	0.854±0.046^a^	0.656±0.038^a^	0.471±0.042
Hyper-BayesCπ1)	0.969±0.022^b^	0.767±0.028^b^	0.542±0.041
Ante-BayesCπ2)	0.934±0.017^ab^	0.551±0.028^a^	0.425±0.022
Ante-hyper-BayesCπ2)	0.977±0.008^b^	0.589±0.028^a^	0.447±0.021

LD, linkage disequilibrium; SNPs, single nucleotide polymorphisms.

1) π represented the proportion of SNPs having no genetics effects on the trait.

2) π represented the proportion of SNPs having no residual genetics effects.

a–b The estimated π values within a column with no common superscript differ significantly (p<0.05); no superscript within a column meant non-significant difference (p>0.05).

**Table 3:** Accuracies and biases of DGVs in the validation population of the common data set from the fifteenth QTL-MAS workshop

Method	Pearson’s correlation	Regression coefficient
Ante-BayesA	0.930	1.044
Ante-BayesB	0.936	1.056
BayesCπ	0.939	1.062
Hyper-BayesCπ	0.941	1.062
Ante-BayesCπ	0.942	1.068
Ante-hyper-BayesCπ	0.942	1.066

DGVs, direct genomic values; QTL-MAS, quantitative trait loci-marker assisted selection.

**Table 4:** Prediction accuracies (mean±standard error) of DGVs in the validation population of mice data over 20 replications

Different SNPs1)	Method	W6W	GSL	BMI	BL
High density	Ante-BayesA	0.455±0.004	0.352±0.005	0.192±0.004	0.234±0.005
	Ante-BayesB	0.453±0.004	0.354±0.005	0.195±0.004	0.238±0.005
	BayesCπ	0.449±0.004	0.352±0.005	0.193±0.004	0.236±0.005
	Hyper-BayesCπ	0.453±0.004	0.353±0.005	0.192±0.004	0.237±0.005
	Ante-BayesCπ	0.448±0.004	0.352±0.005	0.194±0.004	0.236±0.005
	Ante-hyper-BayesCπ	0.452±0.004	0.353±0.005	0.193±0.004	0.237±0.005
Low density	Ante-BayesA	0.416±0.004^a^	0.334±0.005	0.152±0.004	0.199±0.005
	Ante-BayesB	0.363±0.005^b^	0.322±0.006	0.138±0.005	0.188±0.006
	BayesCπ	0.408±0.004^a^	0.334±0.005	0.151±0.004	0.203±0.004
	Hyper-BayesCπ	0.416±0.004^a^	0.334±0.005	0.153±0.004	0.203±0.005
	Ante-BayesCπ	0.411±0.004^a^	0.335±0.005	0.149±0.004	0.203±0.005
	Ante-hyper-BayesCπ	0.415±0.004^a^	0.335±0.005	0.153±0.004	0.203±0.005

DGVs, direct genomic values; SNPs, single nucleotide polymorphisms; W6W, body weight at 6 weeks; GSL, growth slop between 6 and 10 weeks of age; BMI, body mass index; BL, body length.

1) High density, 9266 SNPs; low density, 950 SNPs.

a–b The prediction accuracies within each combination of traits and types of SNPs with no common superscript differ significantly (p<0.05) among six methods; No superscript within each combination of traits and types of SNPs meant non-significant difference (p>0.05).

**Table 5:** Prediction biases (mean±standard error) of DGVs in the validation population of mice data over 20 replications

Different SNPs1)	Method	W6W	GSL	BMI	BL
High density	Ante-BayesA	0.991±0.021	0.982±0.029	0.947±0.039	0.919±0.059
	Ante-BayesB	0.986±0.021	0.998±0.031	0.946±0.044	0.927±0.060
	BayesCπ	0.970±0.018	1.018±0.031	0.975±0.041	0.998±0.064
	Hyper-BayesCπ	1.000±0.022	1.010±0.031	0.992±0.053	0.952±0.061
	Ante-BayesCπ	0.970±0.019	1.016±0.031	0.974±0.044	0.994±0.062
	Ante-hyper-BayesCπ	1.000±0.022	1.010±0.031	0.992±0.052	0.953±0.061
Low density	Ante-BayesA	1.006±0.025	1.009±0.035	0.866±0.044	0.923±0.060
	Ante-BayesB	0.989±0.026	1.046±0.046	0.871±0.061	1.032±0.080
	BayesCπ	0.975±0.024	1.024±0.038	0.899±0.057	1.014±0.065
	Hyper-BayesCπ	1.015±0.027	1.017±0.039	0.924±0.055	0.968±0.070
	Ante-BayesCπ	1.022±0.023	1.032±0.038	0.912±0.071	0.996±0.065
	Ante-hyper-BayesCπ	1.055±0.025	1.028±0.038	0.912±0.055	0.964±0.066

DGVs, direct genomic values; SNPs, single nucleotide polymorphisms; W6W, body weight at 6 weeks; GSL, growth slop between 6 and 10 weeks of age; BMI, body mass index; BL, body length.

1) High density, 9266 SNPs; low density, 950 SNPs.

**Table 6:** π values (mean±standard error) estimated from BayesCπ and extensions of BayesCπ using the reference population of mice data over 20 replications

Different SNPs1)	Method	W6W	GSL	BMI	BL
High density	BayesCπ2)	0.941±0.010^a^	0.415±0.014^a^	0.606±0.033	0.327±0.010^a^
	Hyper-BayesCπ2)	0.661±0.027^b^	0.603±0.020^b^	0.589±0.023	0.595±0.021^b^
	Ante-BayesCπ3)	0.949±0.009^a^	0.485±0.015^c^	0.643±0.030	0.333±0.011^a^
	Ante-hyper-BayesCπ3)	0.694±0.025^b^	0.621±0.016^b^	0.613±0.020	0.591±0.024^a^
Low density	BayesCπ2)	0.626±0.033^a^	0.441±0.020^a^	0.641±0.032^a^	0.334±0.012^a^
	Hyper-BayesCπ2)	0.345±0.019^b^	0.492±0.024^a^	0.524±0.015^b^	0.467±0.014^b^
	Ante-BayesCπ3)	0.673±0.017^a^	0.589±0.013^b^	0.731±0.027^c^	0.427±0.012^b^
	Ante-hyper-BayesCπ3)	0.525±0.009^c^	0.613±0.014^b^	0.590±0.015^ab^	0.572±0.011^c^

SNPs, single nucleotide polymorphisms; W6W, body weight at 6 weeks; GSL, growth slop between 6 and 10 weeks of age; BMI, body mass index; BL, body length.

1) High density, 9,266 SNPs; Low density, 950 SNPs.

2) π represented the proportion of SNPs having no genetics effects on the trait.

3) π represented the proportion of SNPs having no residual genetics effects.

a–c The estimated π values within each combination of traits and types of SNPs with no common superscript differ significantly (p<0.05) among six methods; No superscript within each combination of traits and types of SNPs meant non-significant difference (p>0.05).
