# Supplementary material for: The influence of a first-order antedependence model and hyperparameters in BayesCπ for genomic prediction
Source: Asian-Australas J Anim Sci. 2018 Jul 26;31(12):1863–70. doi: 10.5713/ajas.18.0102 (PMC6212739; doi:10.5713/ajas.18.0102)
Supplement: Supplementary file 1 [file ajas-31-12-1863-supplementary.pdf]

```
!### programmed 2013-12-24(christmas eve)

!!!! re-edit 2017-07-07

program hyper_BayesCpi

   use RANDOMM

  implicit none

     interface

      subroutine correl(x,y,n,corr_r)

       real*8,intent(in)::x(:),y(:)

       real*8 corr_r

       integer n

      end subroutine

      subroutine regress(x,y,n,b,a)

       real*8,intent(in)::x(n),y(n)

       real*8    a,b

       integer n

      end subroutine

         end interface

          integer,parameter :: imode=2      ! 1--- window interaction    2---else

     character(len=100) :: parafile

          character(len=100) :: phenofile,genofile

          integer                      :: nloci,nref,trait_colnum,ncycle,nburning,thin

          integer                      :: gformat ! 1 or 2 or 3

         integer,allocatable:: idstore(:)

          integer                      :: id,sire,dam

          real*8,allocatable:: trait(:),y(:)

          character(len=100) :: temper

          integer,allocatable :: g1(:),g2(:)

          integer,allocatable :: X(:,:)

          integer                          :: idd

          real*8                           :: mu,vare,gvar

          real*8,allocatable    :: g(:)

          real*8                           :: e2,mumean,muvar

          real*8,allocatable    :: ycorr(:)

          integer,allocatable :: sumx(:)

          real*8                           :: gmean,gvar1,mustore,gvarstore,varestore

          real*8,allocatable    :: gstore(:)

          integer                          :: neq

          integer                          :: effloci

     real*8                          :: v1,v0,LH1,LH0

          real*8                           :: alpha

          real*8                           :: pi,sumpi

          real*8                           :: logpi,logpicomp

   ! Iowa

     real*8                          :: p,pq

         ! real*8                          :: va,ve
```

```
            real*8,allocatable    :: ppa(:),ppastore(:)
            real*8                :: sample_mean2pq,mean2pq
            real*8                :: nua,scalea,nuastore,scaleastore
            real*8,allocatable    :: gebv(:)
        integer                   :: i,j,k,cyclen,status
            ! validation population
        character(len=100)    :: phenofile_v,genofile_v
        integer                   :: nval
        real*8                    :: corr_r,reg,inter
        real*8                    :: as,bs
    !!! for nua
      real*8                      :: nua_old,nua_new,lognua
          real*8                  :: LH3,LH4
          real*8                  :: var_nua,alpha1,alphatot
          real*8                  :: sum1,sum2
          integer                 :: m1
open(unit=39,file="log.out",status="replace")
call show(imode,'The BayesCpi program start!',1)
  if(imode==1) then
        write(*,*) "the input filename:"
            read(*,*) parafile
  else
        call getarg(1,parafile)
            parafile=trim(parafile)
  end if
  open(unit=21,file=parafile,status="old");rewind(21)
    read(21,*)
    read(21,*) phenofile
    read(21,*)
    read(21,*) gformat,genofile
    read(21,*)
    read(21,*) nloci
    read(21,*)
    read(21,*) nref
    read(21,*) !!! which column to which trait
    read(21,*) trait_colnum
    read(21,*)
    read(21,*) ncycle
    read(21,*)
    read(21,*) nburning
    read(21,*)
    read(21,*) thin
    read(21,*)
    read(21,*) rseed
  ! read(21,*)
```

```
  ! read(21,*) va
  ! read(21,*)
  ! read(21,*) ve
    read(21,*)
    read(21,*) phenofile_v
    read(21,*)
    read(21,*) genofile_v
close(21)
call show(imode,'All the parameter values ready!',1)
!=======================================================================================================
! read the phenotype records from phenofile
  allocate(idstore(nref))
  allocate(trait(trait_colnum),y(nref))
  open(unit=11,file=phenofile,status="old");rewind(11)
  read(11,*) ! skip phenotype file title
  open(unit=12,file="input_phenotype.out",status="replace")
  k=0
  do while(.true.)
      read(11,*,iostat=status) id,(trait(j),j=1,trait_colnum-1)
            if(status/=0) exit
            k=k+1
            y(k)=trait(trait_colnum-1)
            idstore(k)=id
      write(12,"(i,'      ',f)") idstore(k), y(k)
      if(mod(k,100)==0) then
                write(temper,"(i5,' ','th records finished!')")    k
                call show(imode,temper,0)
            end if
end do
close(11)
close(12)
deallocate(trait)
if(k/=nref) then
      write(*,*) 'the number of individuals for phonefile is inconsistent with the default value!'
      call show(imode,'the number of individuals for phonefile is inconsistent with the default value!',0)
    stop
end if
write(temper,"(i5,' ','th records finished!')")    k
call show(imode,temper,1)
!!! read the genotype from the genofile
!!!!!! three options for genotype format
!!!
open(unit=20,file=genofile,status="old");rewind(20) !!!! no title
open(unit=21,file="input_genotype.out",status="replace")
allocate(X(nref,nloci))
```

```
X=0
k=0
if (gformat==1) then    !!! code 1, 2 for allele in two line --- recode 0,1,2 for genotype
    allocate(g1(nloci),g2(nloci))
    do while(.true.)
        read(20,*,iostat=status) id,id,(g1(j),j=1,nloci)
                 if(status/=0) exit
        read(20,*,iostat=status) idd,idd,(g2(j),j=1,nloci)
                 k=k+1
                 if(id/=idd) then
                     call show(imode,'two line ID of the genofile have error!',1)
                     stop
        end if
                 if(id/=idstore(k))then
                         call show(imode,'Corresponding ID for genotypefile and phenotypefile have error!',1)
                       stop
        end if
        X(k,:)=g1(:)+g2(:)-2
                 write(21,"(i,' ')",advance='NO') id
                 do i=1,nloci
                       write(21,"(i2)",advance='NO') X(k,i)
                   end do
                   write(21,*)
      end do
      deallocate(g1,g2)
elseif(gformat==2) then    !!! code 1, 2 for allele in one line --- recode 0,1,2 for genotype
    allocate(g1(2*nloci))
    do while(.true.)
        read(20,*,iostat=status) id,(g1(j),j=1,2*nloci)
                 if(status/=0) exit
                 k=k+1
                 if(id/=idstore(k))then
                         call show(imode,'Corresponding ID for genotypefile and phenotypefile have error!',1)
                       stop
        end if
                 do i=1, nloci
              X(k,i)=g1(2*i)+g1(2*i-1)-2
                 end do
                 write(21,"(i,' ')",advance='NO') id
                 do i=1,nloci
                       write(21,"(i2)",advance='NO') X(k,i)
                   end do
                   write(21,*)
      end do
      deallocate(g1)
```

```
elseif(gformat==3) then    !!! code 0,1,2 for genotype in one line
      allocate(g1(nloci))
      do while(.true.)
            read(20,*,iostat=status) id,(g1(j),j=1,nloci)
            if(status/=0) exit
                     k=k+1
                     X(k,:)=g1(:)
            write(21,"(i,' ',<nloci>(i2))") id,(X(k,j),j=1,nloci)
                     if(id/=idstore(k))then
                           call show(imode,'Corresponding ID for genotypefile and phenotypefile have error!',1)
                           stop
            end if
      end do
               deallocate(g1)
end if
close(20)
close(21)
  if(k/=nref) then
        call show(imode,'the default nref value unconsistent with the maker file!',0)
        stop
  end if
    pq=0.0D0
    do i=1,nloci
          j=count(X(:,i)==2)
                   k=count(X(:,i)==1)
                   p=real(2*j+k)/real(2*nref)
                   pq=(1.0-p)*p+pq
    end do
    sample_mean2pq=2.0*pq/real(nloci)
  write(39,*) 'sample_mean2pq: ',sample_mean2pq
  call show(imode,'the marker matrix X finished ! ',1)
  !==================================================================================================
  !==================================================================================================
  ! The Gibbs cycles begin
  ! Initialization of g,mu and other arrays
  call show(imode,'Initialization of g,mu and other arrays',1)
  allocate(ycorr(nref),sumx(nloci))
  allocate(gstore(nloci),g(nloci))
  allocate(ppastore(nloci),ppa(nloci)) ! effect ratio for each loci
  !mu=0.1D0
    mu=sum(y)/real(nref); mustore=0.0D0;
    g(:)=0.00D0;gstore(:)=0.0D0
    gvar=0.1D0;gvarstore=0.0D0
    ppa(:)=0.0D0;ppastore(:)=0.0D0
```

```
    pi=0.5D0;sumpi=0.0D0;effloci=0

    vare=0.0D0;varestore=0.0D0

    neq=0

    mean2pq=sample_mean2pq

    !mean2pq=0.5D0

    !nua=4.2D0

    logpi=log(pi)

    logpicomp=log(1.0D0-pi)

    scaleastore=0.0D0; nuastore=0.0D0

    nua=4.2D0

    !scalea=(nua-2.0)*(va/((1.0-pi)*mean2pq*real(nloci)))/nua       ! va- additive variance explained by SNP     scalea=V*S

    scalea=0.3/real(nloci)

    var_nua=0.09D0;alphatot=0.0D0

  ! gvar = va/(real(nloci)*(1.0-pi)*mean2pq)

  ! scalea =nua*((nua-2.0)*gvar/nua)     ! va- additive variance explained by SNP     scalea=V*S

    do i=1,nloci

          sumx(i)=0

                do j=1,nref

                      sumx(i)=sumx(i)+X(j,i)*X(j,i)

                end do

    end do

  ycorr=y-mu

call show(imode,'the Gibbs cycles iterations beginning !',1)

! begin the iteration

outer: do cyclen=1,ncycle

  !    vare=sum(ycorr**2)/chisq(real(nref-2))       ! sample vare from an inverse chi-square posterior Meuwissen(2001)

  !    vare=sum(ycorr**2)/chisq(real(nref+3))     ! R code

  !vare=(sum(ycorr**2)+(nua-2.0)*ve)/chisq(real(nref+nua))     ! habier(2011)

      vare=sum(ycorr**2)/chisq((real(nref)-1.0D0))

               ycorr=ycorr+mu

               mumean=sum(ycorr(:))/real(nref)

               muvar=vare/real(nref)

               mu=mumean+sqrt(muvar)*Stdnorm()

    ! mu=norm(mumean,muvar)       ! Sample the mean from a normal posterior

               ycorr=ycorr-mu

    m1=0

    effloci=0

  ! Sample the g from a normal distribution

do k=1,nloci

      gmean=0.0D0

               do i=1,nref

                   ycorr(i)=ycorr(i)+real(X(i,k))*g(k)

          gmean=gmean+real(X(i,k))*ycorr(i)

               end do
```

```
        v0=real(sumx(k))*vare
                LH0=-0.5D0*(log(v0)+gmean**2/v0)+logpi
                v1=(real(sumx(k)))**2*gvar+v0
                LH1=-0.5D0*(log(v1)+gmean**2/v1)+logpicomp
                alpha=1.0D0/(1.0D0+exp(LH0-LH1))
       !alpha=exp(LH1)*(1.0-pi)/(exp(LH1)*(1.0-pi)+exp(LH0)*pi)
     if(randz()<alpha) then
          gmean=gmean/(real(sumx(k))+vare/gvar)
          gvar1=vare/(real(sumx(k))+vare/gvar)
          g(k)=gmean+sqrt(gvar1)*Stdnorm()
        ! g(k)=norm(gmean,gvar1)
          do i=1,nref
                      ycorr(i)=ycorr(i)-real(X(i,k))*g(k)
                end do
                if(g(k)/=0.0D0) effloci=effloci+1
                ppa(k)=1.0D0
                m1=m1+1
     else
          g(k)=0.0D0
          ppa(k)=0.0D0
    end if
  end do
! for pi
    pi=beta(nloci-effloci+1,effloci+1)
  ! pi=genbet(nloci-effloci+1.0D0,effloci+1.0D0)
    logpi    = log(pi)
    logpicomp =log(1-pi)
    gvar =(nua*scalea+sum(g**2))/chisq(real(effloci)+nua)
    !scalea = nua*((nua-2.0)*(va/((1-pi)*nloci*mean2pq))/nua)
    !gvar =(scalea+sum(g**2))/chisq(real(effloci+nua)
    !!!!!!---------------------------hyper parameter for nua and scalea-----------------------------------------------------------------------------------
    !!!!!---------------------------------------------------------------------------------------------------------------------------------------------------
     !!!-----------------------------------------------------------------------------------------------------------------------------------------------------
     !! Sample s2t from a gamma    distribution(as,bs)
     bs=1.0+nua*real(m1)*(1.0/gvar)/2.0
     as=1.0+nua*real(m1)/2.0
     scalea=gengam(as,bs)
     !!! sample nua uisng a random walk normal MH
     nua_old= nua
     lognua=log(nua)+sqrt(var_nua)*Stdnorm()
     nua_new= exp(lognua)
     !LH1=real(nloci)*(nua_new/2.0*(log(nua_new)+log(scalea)-log(2.0))-log(GA(nua_new/2.0)))-(1.0+nua_new/2.0)*sum(log(gvar))-(nua_new*scalea/2.0)*sum(1.0/gvar)
     !LH0=real(nloci)*(nua_old/2.0*(log(nua_old)+log(scalea)-log(2.0))-log(GA(nua_old/2.0)))-(1.0+nua_old/2.0)*sum(log(gvar))-(nua_old*scalea/2.0)*sum(1.0/gvar)
     LH4=real(m1)*(nua_new/2.0*(log(nua_new)+log(scalea)-log(2.0))-log(GA(nua_new/2.0)))-(1.0+nua_new/2.0)*real(m1)*log(gvar)-(nua_new*scalea/2.0)*real(m1)*(1.0/gvar)
     LH3=real(m1)*(nua_old/2.0*(log(nua_old)+log(scalea)-log(2.0))-log(GA(nua_old/2.0)))-(1.0+nua_old/2.0)*real(m1)*log(gvar)-(nua_old*scalea/2.0)*real(m1)*(1.0/gvar)
```

```
      LH4=LH4-2.0*log(1+nua_new)+log(nua_new)

      LH3=LH3-2.0*log(1+nua_old)+log(nua_old)

      alpha1=exp(LH4-LH3)

      alpha1=min(alpha1,1.0)

      !accept and rejection rules

      if(alpha1<1.0) then

          if(alpha1>randz()) then

                  nua=nua_new

              else

                  nua=nua_old

          end if

      else

              nua=nua_new

      end if

       alphatot=alphatot+alpha1

       if(cyclen<=int(nburning*2/3).and.mod(cyclen,10)==0) then

              alphatot=alphatot/10.0

              if(alphatot>0.8) var_nua=1.2*var_nua

          if(alphatot<0.2) var_nua=0.7*var_nua

              alphatot=0.0

       end if

   ! write(*,*) nua,scalea

   ! if(cyclen==20) stop

!!!------------------------------------------------------------------------------------------------------------------------------------------------------------

    ! caculate the snp effect and var for mean

    if(cyclen>nburning.and.mod(cyclen,thin)==0) then

        neq=neq+1

        gstore=gstore+g

            gvarstore=gvarstore+gvar

            mustore=mustore+mu

            sumpi=sumpi+pi

            ppastore=ppastore+ppa

            varestore=varestore+vare

        nuastore=nuastore+nua

        scaleastore=scaleastore+scalea

    end if

   if(mod(cyclen,100)==0) then

       write(temper,"(i6,' th cycle finished.')") cyclen

           call show(imode,temper,1)

   end if

end do outer

   deallocate(y)

   deallocate(ycorr,sumx)
```

```
  g=gstore/real(neq)
  gvar=gvarstore/real(neq)
  ppa=ppastore/real(neq)
  mu=mustore/real(neq)
  sumpi=sumpi/real(neq)
  vare=varestore/real(neq)
  nua=nuastore/real(neq)
  scalea=scaleastore/real(neq)
  deallocate(gstore,ppastore)
call show(imode,'the gibbs cycle finished and all result stored!',1)
!=======================================================================================================================
! output the result
  open(unit=23,file="snpeff.out",status="replace")
    write(23,*)'pi:',sumpi
    write(23,*)'snpvar:',gvar
    write(23,"('effect','                ','level','    ','gi','         ','effect_ratio')")
    write(23,"('1','       ','1','          ',f)") mu
    do i=1,nloci
          write(23,"('2',' ',i6,'          ',f,'            ',f)")i,g(i),ppa(i)
    end do
close(23)
allocate(gebv(nref))
  do i=1,nref
        gebv(i)=mu
    do j=1,nloci
          gebv(i)=gebv(i)+real(X(i,j))*g(j)
    end do
end do
open(unit=24,file="GEBV.out",status="replace")
write(24,"('ID','         ','gebv')")
do i=1,nref
  write(24,"(i5,'         ',f)") idstore(i),gebv(i)
end do
close(24)
open(unit=24,file="Ve_dfa_scalea.out",status="replace")
write(24,"(A5,' ',f)") 'Ve:',vare
write(24,"(A5,' ',f)") 'dfa:',nua
write(24,"(A8,' ',f)") 'scalea:',scalea
close(24)
deallocate(idstore,ppa)
deallocate(X,gebv)
!=======================================================================================================================
!=======================================================================================================================
!output validation population results
! read records from phenofile
```

```
  allocate(idstore(60000))
  allocate(trait(trait_colnum),y(60000))
  open(unit=11,file=phenofile_v,status="old");rewind(11)
  read(11,*) !!!! skip title for phenotype file
  k=0
  do while(.true.)
      read(11,*,iostat=status) id,(trait(j),j=1,trait_colnum-1)
              if(status/=0) exit
              k=k+1
              y(k)=trait(trait_colnum-1)
              idstore(k)=id
end do
close(11)
deallocate(trait)
write(temper,"(i7,' ','th records for validation population finished!')")    k
call show(imode,temper,1)
nval=k
!!!!!! three options for genotype format
!!! get X matrix
open(unit=20,file=genofile_v,status="old");rewind(20) !!!! no title
allocate(X(nval,nloci))
X=0
k=0
if (gformat==1) then    !!! code 1, 2 for allele in two line --- recode 0,1,2 for genotype
    allocate(g1(nloci),g2(nloci))
    do while(.true.)
        read(20,*,iostat=status) id,id,(g1(j),j=1,nloci)
                if(status/=0) exit
        read(20,*,iostat=status) idd,idd,(g2(j),j=1,nloci)
                k=k+1
                if(id/=idd) then
                    call show(imode,'two line ID of the genofile have error!',1)
                    stop
        end if
                if(id/=idstore(k))then
                        call show(imode,'Corresponding ID for genotypefile and phenotypefile have error!',1)
                       stop
        end if
        X(k,:)=g1(:)+g2(:)-2
    end do
      deallocate(g1,g2)
elseif(gformat==2) then    !!! code 1, 2 for allele in one line --- recode 0,1,2 for genotype
    allocate(g1(2*nloci))
    do while(.true.)
        read(20,*,iostat=status) id,(g1(j),j=1,2*nloci)
```

```
                    if(status/=0) exit
                    k=k+1
                    if(id/=idstore(k))then
                            call show(imode,'Corresponding ID for genotypefile and phenotypefile have error!',1)
                          stop
          end if
                    do i=1, nloci
                 X(k,i)=g1(2*i)+g1(2*i-1)-2
                    end do
      end do
        deallocate(g1)
  elseif(gformat==3) then    !!! code 0,1,2 for genotype in one line
      allocate(g1(nloci))
        do while(.true.)
              read(20,*,iostat=status) id,(g1(j),j=1,nloci)
              if(status/=0) exit
                        k=k+1
                        X(k,:)=g1(:)
                        if(id/=idstore(k))then
                              call show(imode,'Corresponding ID for genotypefile and phenotypefile have error!',1)
                              stop
              end if
        end do
                 deallocate(g1)
  end if
  close(20)
    if(k/=nval) then
          call show(imode,'the default nval value unconsistent with the maker file!',0)
          stop
    end if
    !!! calculate GEBV for validation population
    allocate(gebv(nval))
    gebv=0.0
    do i=1,nval
        gebv(i)=mu
        do j=1,nloci
              gebv(i)=gebv(i)+real(X(i,j))*g(j)
        end do
    end do
    call correl(gebv,y(1:nval),nval,corr_r)
    call regress(gebv,y(1:nval),nval,reg,inter)
open(unit=24,file="GEBV_validation.out",status="replace")
write(24,*)'Accuracy of genomic prediction:'
write(24,*)'Corr:', corr_r
write(24,*)'Reg:', reg, 'Inter:', inter
```

```
write(24,"('ID','          ','gebv','    ','TBV')")
do i=1,nval
    write(24,"(i7,'        ',f,'             ',f)") idstore(i),gebv(i),y(i)
end do
close(24)
deallocate(idstore,gebv,g,X,y)
call show(imode,'the program BayesCpi run very good!',1)
  stop
end program
  !===============================================================================
             ! calculate correlation between two samples
          subroutine correl(x,y,n,corr_r)
              real*8,intent(in)::x(:),y(:)
              real*8 corr_r
              integer n
              real*8 sum_xy,sum_x,sum_xx,sum_y,sum_yy,tmp_a
              sum_xy=0.0;sum_x=0.0;sum_xx=0.0;sum_y=0.0;sum_yy=0.0
              do i=1,n
                    sum_xy= sum_xy+x(i)*y(i)
                    sum_x = sum_x+x(i)
                    sum_xx= sum_xx+x(i)**2
                    sum_y=sum_y+y(i)
                    sum_yy=sum_yy+y(i)**2
              enddo
              tmp_a=sqrt(n*sum_xx-sum_x**2)*sqrt(n*sum_yy-sum_y**2)
              corr_r=(n*sum_xy-sum_x*sum_y)/tmp_a
          end subroutine
  !===============================================================================
  ! calculate y=a+bx for two samples
          subroutine regress(x,y,n,b,a)
              real*8,intent(in)::x(n),y(n)
              real*8    a,b
              integer n
              real*8 sum_xx,sum_xy,aver_x,aver_y
              sum_xy=0.0;sum_xx=0.0;aver_x=0.0;aver_y=0.0
                     aver_x=sum(x(:))/real(n)
                     aver_y=sum(y(:))/real(n)
              do i=1,n
                    sum_xy= sum_xy+(x(i)-aver_x)*(y(i)-aver_y)
                    sum_xx= sum_xx+(x(i)-aver_x)**2
            end do
              b=sum_xy/sum_xx
              a=aver_y-b*aver_x
        end subroutine
```
